# Highly luminescent polyethylene glycol-passivated graphene quantum dots for light emitting diodes†

**DOI:** 10.1039/d0ra02257h

**Published:** 2020-07-22

**Authors:** Hyun Jun Kim, Chung Kyeong Lee, Jin Gwan Seo, Soon Jik Hong, Gian Song, Junghoon Lee, Changui Ahn, Dong Ju Lee, Sung Ho Song

**Affiliations:** Division of Advanced Materials Engineering, Kongju National University Cheonan Chungnam 32588 Republic of Korea; Division of Chemical Engineering, Dongseo University 47, Jurye-ro, Sasang-gu Busan 47011 Republic of Korea; Engineering Ceramic Center, Korea Institute of Ceramic Engineering and Technology Icheon Cyeonggi 17303 Republic of Korea; Department of Advanced Materials Engineering, Chungbuk National University Cheonju Chungbuk 28644 Republic of Korea

## Abstract

The emergence of fluorescent graphene quantum dots (GQDs) is expected to enhance the usefulness of quantum dots (QDs), in terms of their unique luminescence, photostability, low toxicity, chemical resistance, and electron transport properties. Here we prepared blue-photoluminescent polyethylene glycol GQDs (PEG-GQDs) through PEG surface passivation. The photoluminescence (PL) quantum yield (QY) of PEG-GQDs with 320 nm excitation was about 4.9%, which was higher than that of pure GQDs. The as-fabricated PEG-GQDs with high QY were then used as light-emitting diode (PGQD-LED) emitters, in which the GQDs were incorporated into polymeric host layers in a multilayer electroluminescent device; blue emission with a luminance exceeding 800 cd m^−2^ was achieved, thus demonstrating the potential of PEG-GQDs as emitters in electroluminescence applications. Furthermore, the fluorescence mechanism of PEG-GQDs was investigated and proved that the origin of strong fluorescence of PEG-GQDs is associated with the luminescence from intrinsic states. The highly fluorescent PEG-GQDs will allow new devices, such as multicolor LEDs, to be developed with extraordinary properties, by tailoring the intrinsic and extrinsic states.

## Abstract 2

We prepared blue-photoluminescent polyethylene glycol GQDs (PEG-GQDs) through PEG surface passivation. The PL intensity was stronger than that of pristine GQDs. These were then used as LED emitters and the fluorescence mechanism was investigated.

## Graphical abstract

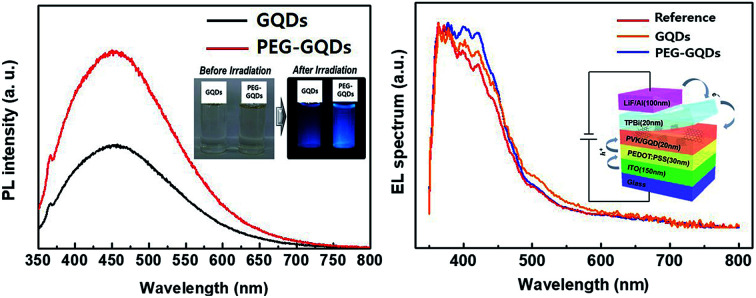

## 1 Introduction

The large optical absorptivity and widely tunable band gap of graphene make it an attractive material for optical and optoelectronic devices.^1,2^ Recently, graphene quantum dots (GQDs), with a size of only several nanometers, have demonstrated novel physical and chemical properties due to quantum confinement^3,4^ and edge effects;^5^ these properties have been expected to be especially useful in advancing GQD technology for new applications. Especially, GQDs with strong fluorescence are expected to serve as a next-generation nanomaterial and a promising alternative to fluorescent semiconductor quantum dots (QDs), which are composed of toxic and expensive heavy metals.^6,7^ In addition to stable photoluminescence (PL), GQDs are increasingly gaining attention due to their chemical inertness and excellent biocompatibility, which makes them a potential candidate for bio-sensor, bio-imaging, and photovoltaic applications.^8–13^

To date, GQDs have been fabricated by various methods and techniques involving organic synthesis transformation,^14^ electron beam lithography,^15^ C_60_ molecules,^16^ electrochemical processing,^17^ and hydrothermal cutting.^18,19^ Despite some success in GQD synthesis, these methodologies showed several limitations. For example, the use of electron beam lithography is limited by the requirement for special equipment and extremely expensive raw materials, as well as a low yield. Solution-based cleavage methods required a series of chemical treatments that typically take several days and involve various chemical reagents.

In this study, GQDs with blue emission at 430 nm were fabricated on a large scale from graphite intercalation compounds (GICs). The proposed method is cost-effective and eco-friendly, because intercalation from potassium-sodium tartrate (KNaC_4_H_4_O_6_·4H_2_O) and the dissolution process in water are performed without surfactants or chemical solvents. However, defects and oxygen functional groups inevitably form at the edges and on the basal plane of the GQDs, which makes it difficult to achieve strong fluorescence and interferes with practical application due to a low quantum yield (QY). Therefore, surface passivation of GQDs with various polymer or organic molecules is an important pathway for further improving the fluorescence intensity of the GQDs and tailoring the fluorescence *via* defect formation. Also, to suppress aggregation and PL quenching, an appropriate passivation of the GQDs is needed, due to their high solubility in organic solvents. Dai *et al.*^20^ demonstrated the intrinsic fluorescence of the graphene oxide quantum dots (GOQDs) passivated with polyethylene glycol (PEG) in both the near-infrared and visible regions. Shen *et al.*^21^ reported a method to fabricate surface-passivated the GQDs with PEG *via* reduction of the GOQDs using hydrazine hydrate. However, due to the strong acid treatment, this process had increased the oxygen content and defect formation, even with polymer passivation, thus preventing a fundamental understanding of the fluorescence properties and their practical application.

Here, we introduce a simple method to fabricate the PEG-GQDs *via* a solvothermal reaction, using GQDs and PEG as starting materials. The GQDs were synthesized by a novel GIC-based process with KNaC_4_H_4_O_6_·4H_2_O and graphite in the hydrothermal reactor. We also examined the origin of the PL mechanism. Generally, the fluorescence of GQDs has two spectral states: the intrinsic state from isolated sp^2^ nano-domains and an extrinsic state associated with defects. Interestingly, these GQDs with PEG showed strong blue emission, which was attributed to the significant effect of the intrinsic states due to a lower defect concentration in the GQDs. To confirm the performance of the as-fabricated PEG-GQDs for optoelectronic applications, we directly employed them as emitters in LEDs. The PEG-GQDs LEDs showed a maximum luminance of 800 cd m^−2^. Thus, our results suggest that PEG-GQDs allowed control over the fundamental properties of the materials through their intrinsic and extrinsic effects. The proposed approach is expected to promote the development of new devices with extraordinary properties and functions for a wide range of bioimaging and optoelectronic applications.

## 2 Experimental

### 2.1. Materials

Graphite powder was obtained from Bay Carbon, Inc. (SP-1 graphite powder). Potassium sodium tartrate was obtained from Sigma-Aldrich.

### 2.2. Preparation of GQDs

The GQDs were fabricated through GICs with potassium sodium tartrate at low temperature. The potassium sodium tartrate (200 mg) and graphite (20 mg, SP-1, Bay Carbon Inc.) were mixed and grinded in the mole fraction ratio. Then, the grinded homogeneous mixtures are reacted to the autoclave vessel at 250 °C for 12 hours. The manufactured GICs are exfoliated in water and then well-defined GQDs are produced. The sizes of the GQDs were controlled by filtration methods, and the dialyzed in dialysis tubing to remove the remaining salts. Finally, the GQDs were obtained after dried under vacuum for several days.

### 2.3. Preparation of PEG-GQDs

The GQDs in water was mixed with PEG-bisamine of 2000 molecular weight in a ratio of 2 mg PEG per ml of solution. The mixture was reacted at 110 °C with stirring for 24 hour in order to ensure fully functionalization. After cooling, the dialysis process was repeated to remove any functionalized PEG.

### 2.4. Characterization of GQDs and PEG-GQDs

UV-vis spectra (UV-3101PC spectrometer), fluorescence spectra (PerkinElmer LS 55 luminescence spectrometer), X-ray photoelectron spectroscopy (XPS, Sigma Probe, Al Kα), transmission electron microscopy (TEM, Tecnai G2 F30) analyses were conducted. Morphology of GQDs was analysed using an atomic force microscope (AFM, SPA400, SII, Japan) in tapping mode under ambient conditions. TEM samples were prepared by drying a droplet of the GQDs suspensions on a carbon grid. Raman spectra were obtained from 1200 to 3000 cm^−1^ using a Raman spectrometer (LabRAM HR UV/Vis/NIR, excitation at 514 nm). The FT-IR spectrum was measured using a FT-IR-4100 type-A FT-IR spectrometer with pure KBr as the background from 1000 and 3000 cm^−1^.

The photoluminescence (PL) measurements were carried out using a 325 nm He–Cd continues-wave (CW) laser, a monochromatic light from a 300 W-xenon lamp, and UV spectrometers (Maya2000, Ocean Optics, USA) as a PL detector at room temperature. The PL excitations were measured by monochromatic light from a 300 W xenon lamp and a high-sensitive photomultiplier tube as a PL detector. A mode-locked femto-second pulsed Ti:sapphire laser (Coherent, Chameleon Ultra II) system was used as an excitation source, and the diverse wavelengths of the pulsed Ti:sapphire laser were employed.

### 2.5. Device fabrication

The GQDs-LED having the structure of ITO (150 nm)/poly(3,4-ethylenedioxythiophene):poly(styrene sulfonate) (PEDOT:PSS) (30 nm)/3 wt% GQDs (or PEG-GQDs) doped poly(*N*-vinylcarbazole) (PVK)/2,2′,2′′-(1,3,5-benzinetriyl)-tris(1-phenyl-1-*H*-benzimidazole) (TPBi) (60 nm)/lithium fluoride (LiF) (1 nm)/Al (100 nm) was fabricated. Glass substrates pre-coated with 150 nm thick ITO were cleaned in an ultrasonic bath using soapy water, DI water, acetone and isopropyl alcohol and treated by air plasma using a plasma cleaner (PDC-32G, Harrick Plasma). The conducting polymer PEDOT:PSS (Baytron AI4083, H.C. Starck) was spun (2500 rpm for 30 s) on the cleaned substrates. The PVK doped with GQDs (or PEG-GQDs) (3 wt%) or PVK layers were spin-coated on top of the PEDOT:PSS layers. The samples were then loaded into a thermal evaporation chamber (HS-1100, Digital Optics & Vacuum), which was enclosed by another glove box connected in tandem with the aforementioned glove box. The subsequent organic layers and metal layers were successively deposited under high vacuum (∼1 × 10^−6^ Torr). The current density–luminance–voltage characteristics of fabricated devices were evaluated using a source-measure unit (Keithley 2400) and a calibrated photodiode (FDS100, Thorlab). The electroluminescence spectra were obtained with a fiber optics spectrometer (EPP2000, StellarNet). All of the measurement process was conducted in the inert atmosphere.

## 3 Results and discussion

**Fig. 1:**
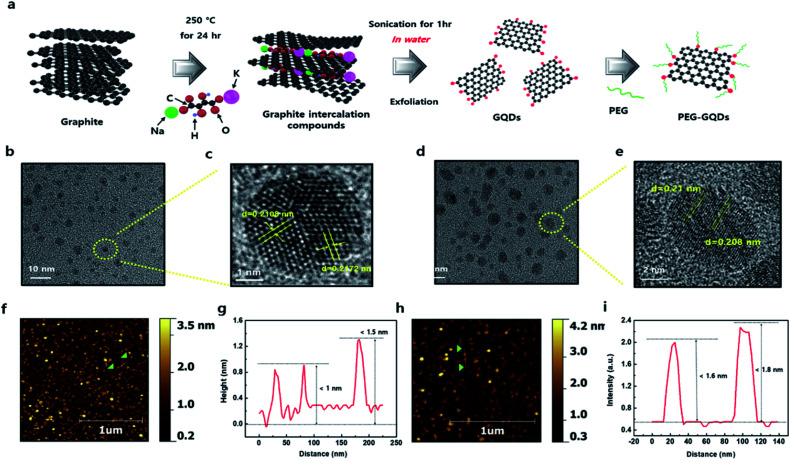
Schematic diagram showing the overall processing and characterization of graphene quantum dots (GQDs). (a) Schematic illustration of the preparative strategy of polyethylene glycol-passivated GQDs (PEG-GQDs). (b) High-resolution (HR-TEM) image of GQDs. (c) HR-TEM image of the GQDs in the yellow circle in (b). (d) HR-TEM image of PEG-GQDs. (e) HR-TEM image of the PEG-GQDs in the yellow circle in (d). The HR-TEM images of all samples indicate the high crystallinity of GQDs, with a lattice spacing of 0.208 nm. (f) Atomic force microscopy (AFM) image of GQDs on a mica substrate. (g) Thickness of GQDs on a mica substrate. (h) AFM image of GQDs on a mica substrate. (i) Thickness of GQDs on a mica substrate.

The synthesis process of PEG-GQDs is illustrated in Fig. 1a. The GICs were synthesized with graphite and KNaC_4_H_4_O_6_·4H_2_O at low temperature for minimal oxidation of the basal plane of GQDs. Fig. S1† shows the phase transition of intercalated graphite intercalation in the X-ray diffraction (XRD) pattern. The main peaks of GICs (∼6.02 Å) and graphite (∼3.34 Å) appeared at 14.1° and 26.4°, respectively, indicating highly ordered GICs with the insertion of potassium metal and the tartrate chain (Fig. S1†). In contrast to conventional KC8 GICs, the GICs here were also stable in air, due to the incorporation of tartrate groups. The PEG-GQDs were then synthesized using a solvothermal reaction with PEG surface passivation *via* the amine bond. In Fig. 1b, c and S2,† a high-resolution transmission electron microscopy (HR-TEM) images of the GQDs show a highly crystalline structure with a lattice spacing of 0.2172 nm, consistent with previous reports.^22,23^ Unlike the GQDs, the size of PEG-GQDs increased to over 5 nm with no change in the lattice spacing of 0.21 nm, as shown in Fig. 1d, e and S3.† Atomic force microscopy (AFM) images of the GQDs and PEG-GQDs morphologies are shown in Fig. 1f–i and S4;† the thickness of the GQDs was <1.5 nm and that of the PEG-GQDs was within 1.8 nm by wrapping of the GQDs with PEG molecules. In Fig. S5,† the GQD thickness was mainly between 1.0 and 4.0 nm, and the topological height was within the range of 0.5 to 1.5 nm. The diameter of the PEG-GQDs was mainly between 5.0 to 7.0 nm, and their topological height was 2.0 to 4.0 nm (Fig. S6†).

**Fig. 2:**
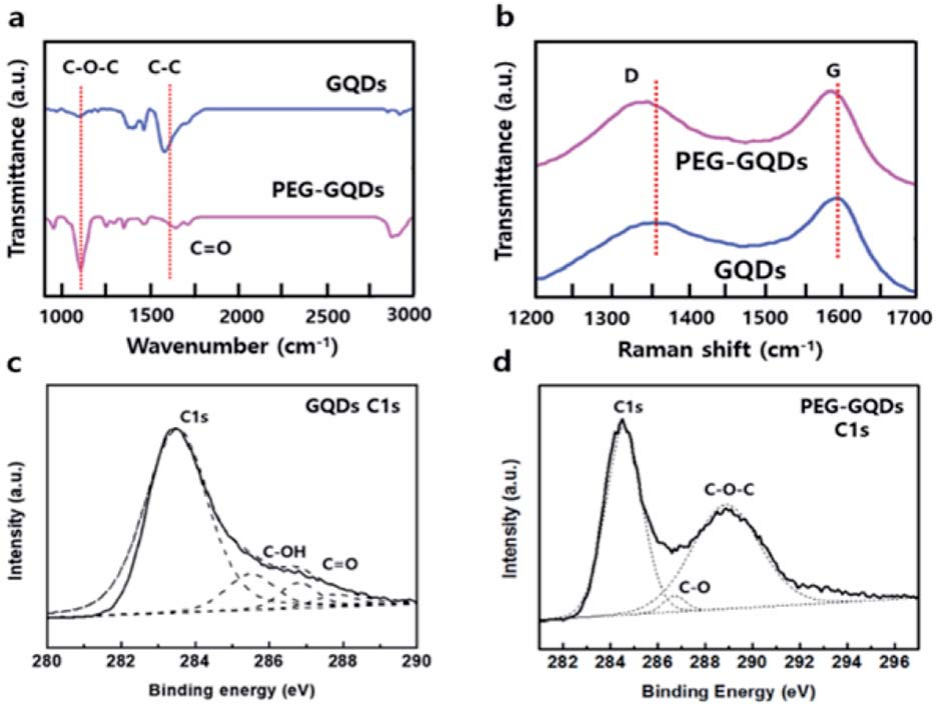
Comparison of GQDs and PEG-GQDs properties. (a) Fourier transform infrared (FT-IR) spectra of GQDs and PEG-GQDs. (b) Raman spectra of GQDs and PEG-GQDs. (c) High-resolution X-ray photoelectron spectroscopy (XPS) C 1s spectra of GQDs. (d) High-resolution XPS C 1s spectra of PEG-GQDs.

Fourier transform infrared spectroscopy (FT-IR) analysis of GQDs and PEG-GQDs revealed peaks associated with the C–C bond (1524 cm^−1^) in both samples, as shown in Fig. 2a. The epoxide peak at 1024 cm^−1^ was completely absent from GQDs; however, the peak appeared prominently in PEG-GQDs. Generally, epoxy groups on the surface of GQDs serve as chemically reactive sites for the rupture of underlying C–C bonds; additionally, the epoxy groups usually induce non-radiative recombination of localized electron–hole pairs. Raman spectra in Fig. 2b provide convincing evidence for the formation of high-quality GQDs. The PEG-GQDs displayed a disorder (D) band at 1356 cm^−1^ and a sp^2^-bond with C atoms (G) band at 1594 cm^−1^; the D to G ratio (*I*_D_/*I*_G_) was 0.873, which was smaller than that of the GQDs (1.102). These results support that the synthesized PEG-GQDs have a pure sp^2^ carbon crystalline structure with fewer defects with PEG passivation, which is extremely important for understanding the optical PL origin of the GQDs and GOQDs. Furthermore, the G and D peaks of PEG-GQDs were shifted slightly, which indicates that PEG may have electron-donating properties with regard to GQDs.^24^ The X-ray photoelectron spectroscopy (XPS) spectra in Fig. 2c and S7† show a dominant sp^2^ carbon peak at 284.5 eV, with negligible oxygenous peaks caused by tartrate functional groups of the GQDs. In contrast, the XPS spectra of PEG-GQDs indicated the presence of numerous oxygen functional groups of carbon atoms for C–C (284.5 eV), C–OH (286.6 eV), and C–O–C (288.2 eV) bonds through PEG passivation (Fig. 2d and S8†).

**Fig. 3:**
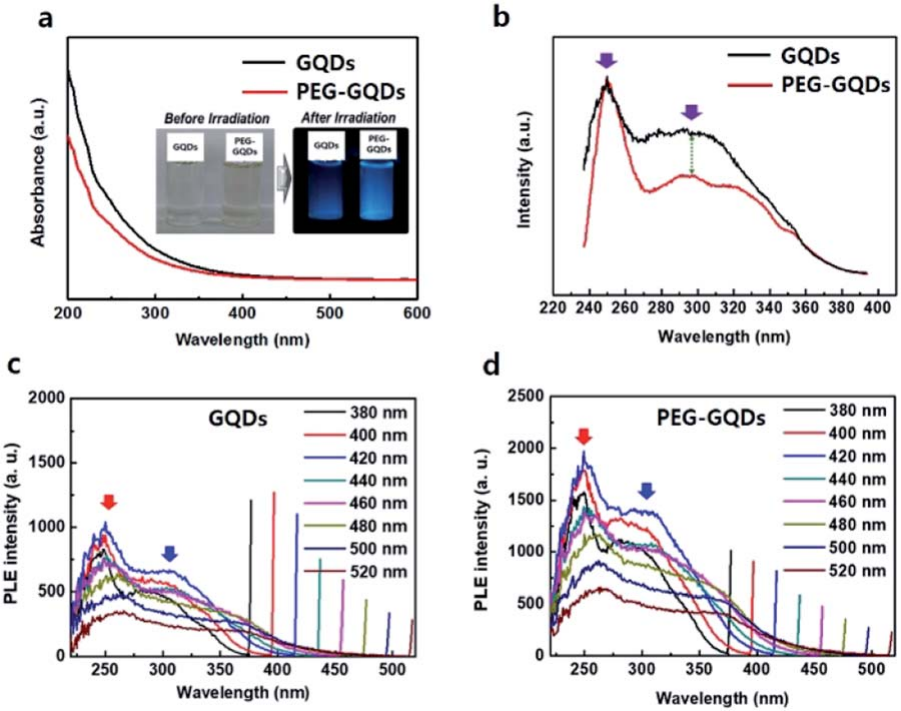
Optical properties of GQDs and PEG-GQDs. (a) Ultraviolet-visible (UV-vis) absorbance of GQDs (black) and PEG-GQDs (red). (b) Excitation wavelength (*λ*_ex_)-dependent photoluminescence (PLE) spectra of GQDs and PEG-GQDs. (c) PLE spectra of GQDs with respect to *λ*_ex_. (d) PLE spectra of PEG-GQDs with respect to *λ*_ex_.

In Fig. 3a, ultraviolet-visible (UV-vis) spectra of GQDs and PEG-GQDs exhibited a typical absorption peak at ∼260 nm for the GQDs and PEG-GQDs. This peak is assigned to the π–π* transition of aromatic sp^2^ domains. For the PEG-GQDs, however, the absorption intensity was lower than that of GQDs. To further clarify the mechanisms of luminescence in GQDs and PEG-GQDs, the excitation wavelength (*λ*_ex_)-dependent PL (PLE) was measured at the center of intrinsic and extrinsic PL peaks of both GQDs and PEG-GQDs. In GQDs and PEG-GQDs, the PLE spectra showed a sharp peak at ∼250 nm, originating from the π–π* transition in sp^2^ domains; additionally, a broad shoulder was observed near 300 nm, related to the n–π* transition for the oxygen defects shown in Fig. 3b. For the PEG-GQDs samples, the intensity of the PLE peak at ∼250 nm was higher than that of GQDs; thus, PEG-GQDs displayed fewer defects due to PEG passivation. Fig. 3c and d shows the PLE of GQDs and PEG-GQDs as a function of *λ*_ex_. As *λ*_ex_ increased, the sharp PLE peak (red arrow) at 250 nm decreased, and the PLE peak at 300 nm (blue arrow) increased in all samples. These results indicate that for *λ*_ex_ < 460 nm, both intrinsic and extrinsic states can be excited, whereas when *λ*_ex_ > 460 nm, excitation occurs only from the extrinsic states associated with the defects (Fig. 3c and d). Furthermore, the PLE peaks of PEG-GQDs were more intense than those of GQDs for *λ*_ex_ ranging from 266 to 520 nm, due to the existence of defects in the PEG-GQD samples (Fig. 3b and d).

**Fig. 4:**
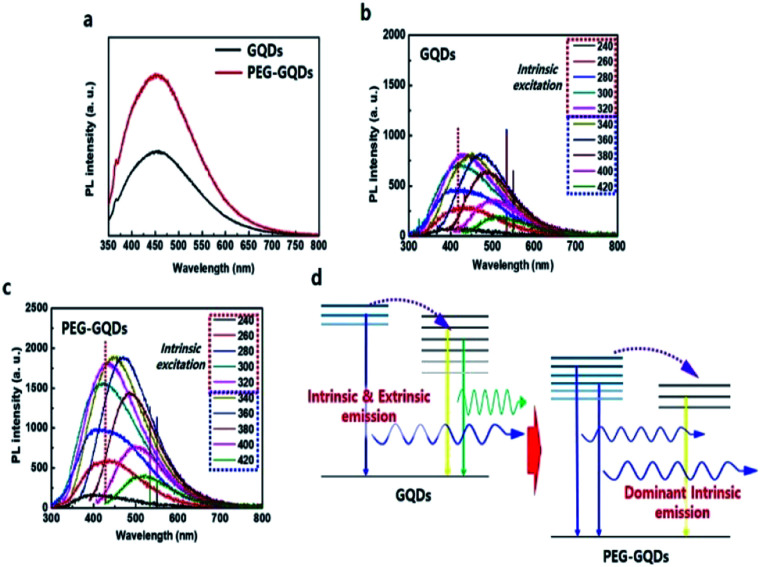
Optical properties of GQDs and PEG-GQDs. (a) PL spectra of GQDs (red) and GOQDs (green) under excitation at 310 nm of monochromatic light from a xenon lamp. (b) PL spectra of GQDs with respect to *λ*_ex_. (c) PL spectra of PEG-GQDs with respect to *λ*_ex_. (d) Modelling of luminescent mechanism of GQDs and PEG-GQDs.

Under 310 nm excitation from a xenon lamp, the PL spectra of GQDs and PEG-GQDs exhibited a strong peak at ∼430 nm (blue emission), as shown in Fig. 4a; the PL intensity of PEG-GQDs was stronger than that of pristine GQDs. The PL properties of GQDs and PEG-GQDs were investigated under different *λ*_ex_, as shown in Fig. 4b and c; a higher PL intensity of PEG-GQDs was observed compared with GQDs, at all *λ*_ex_. Also, according to the *λ*_ex_, the centers of the emission profiles of GQDs were more red-shifted than those of PEG-GQDs, which indicates that the GQDs have more extrinsic state emission originating from defects, as shown in Fig. 4c, d and S9.† Comparing the optical properties of GQDs and PEG-GQDs, our results strongly suggest that the strong PL in PEG-GQDs originate from the transition of their intrinsic states derived from pure sp^2^-bonding in high-quality GQDs with blue spectral output.^25^ The PL mechanism of both GQDs and PEG-GQDs is displayed in Fig. 4d; in both samples, while optically excited carriers with *λ*_ex_ < 320 nm transfer from intrinsic to extrinsic states, excited carrier transfer does not occur for *λ*_ex_ > 320 nm, because only the extrinsic states are excited. Furthermore, as shown in Fig. 4d, the blue emission of PEG-GQDs, which originates from the intrinsic states of sp^2^ domains, is stronger than that of GQDs due to the lower number of defects associated with PEG passivation.

**Fig. 5:**
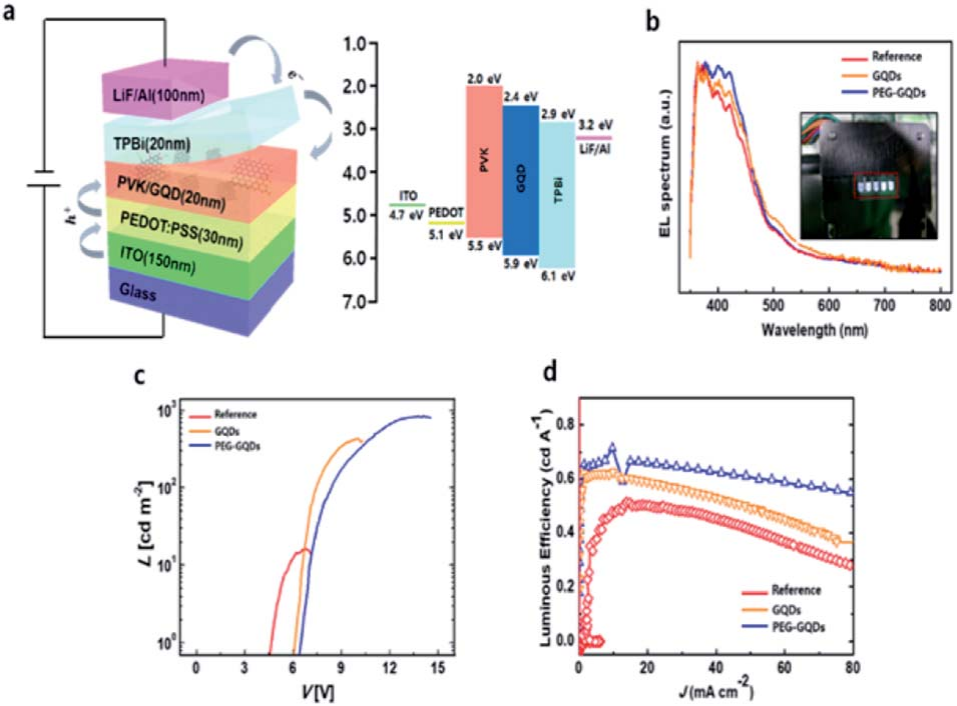
Device characteristics of a GQDs-LED. (a) Schematic illustration of the GQDs-LED structure and the corresponding band diagram. (b) Electroluminescence (EL) spectra of the GQDs-LED and PEG-GQDs LED. Inset: EL image of the LED, consisting of five emitting areas. (c) Current density–voltage (*J*–*V*) and luminance–voltage (*L*–*V*) characteristic curves for the reference, GQDs-LED, and PEG-GQDs LED. (d) Luminous efficiency and emission spectra of the devices.

Finally, the QYs for GQDs and PEG-GQDs were measured using an absolute PL QY measurement system under the same conditions as in Fig. S10.† The value of the QY of PEG-GQDs was 4.9%, whereas the GQDs had a QY of only 4.3%, as shown in Fig. S10.† In virtue of our PEG-GQDs having a relatively high QY, we applied the PEG-GQDs to LED devices. The GQDs and PEG-GQDs are also uniformly dispersed in PVK host matrix in Fig. S11.†Fig. 5a shows the GQD-based LED device (GQD-LED) structure consisting of ITO/PEDOT:PSS/PVK:GQDs (or PEG-GQDs)/TPBi/LiF/Al and the corresponding energy diagram, in which PEDOT:PSS, PVK, and TPBi refer to the poly(3,4-ethylenedioxythiophene):poly(styrene sulfonate), poly(*N*-vinylcarbazole) (PVK), and 2,2′,2′′-(1,3,5-benzinetriyl)-tris(1-phenyl-1-*H*-benzimidazole), respectively, shown in Fig. S12.†Fig. 5b shows the EL spectrum of LED devices according to *λ*_ex_. All devices showed a strong peak at ∼400 nm (blue emission), in which the results were identical to the PL spectrum. Fig. 5c shows the current density–voltage (*J*–*V*) and luminance–voltage (*L*–*V*) characteristic curves of LEDs fabricated with GQDs and PEG-GQDs. The turn-on voltage (*V*_t_) was as large as 6.5 V for the reference device containing PVK with PEG-GQDs; however, *V*_t_ decreased to ∼6.0 V for the devices with PVK:GQDs (3 wt%) due to a reduction in conductivity *via* passivation with long-chain PEG. Our results indicated that GQDs and PEG-GQDs may provide additional carrier transport/injection pathways, resulting in an enhancement in the overall current density. In turn, this would increase the chance of radiative recombination of GQDs and PEG-GQDs. Luminance exceeding 800 cd cm^−2^ was measured for a device with 3.0 wt% PEG-GQDs at 15 V, through improved dispersibility in PVK and the high QY of PEG-GQDs. Fig. 5d compares the luminous efficiencies and emission spectra of the devices. A luminous efficiency of 0.61 cd A^−1^ was obtained for the 3.0 wt% PEG-GQDs device at a current density of 1 mA cm^−2^, while the reference device without GQDs showed an efficiency of only 0.49 cd A^−1^. This increase in luminous efficiency is attributed to the fluorescent emission of PEG-GQDs dispersed in the devices, which can be identified in the emission spectrum of the PEG-GQDs-containing device and is clearly different from that of the pure PVK sample.

## 4 Conclusions

In this study, we developed a novel solvothermal method for synthesizing PEG-GQDs with high blue PL efficiency. An investigation of the carrier dynamics revealed that the blue emission of PEG-GQDs was stronger than that of GQDs, due to a higher concentration of intrinsic states of the sp^2^ domain *via* suppression of defect formation through PEG passivation. Furthermore, the PL QY of the PEG-GQDs was significantly larger than that of GQDs. LED devices produced using the as-fabricated PEG-GQDs as the emissive material demonstrated enhanced EL performance relative to the LEDs manufactured with GQDs. A luminance >800 cd cm^−2^ was measured for a device with 3.0 wt% PEG-GQDs at 15 V. This is higher than previously reported value on EL devices with PEG-GQDs through improved dispersibility in PVK and the high QY of PEG-GQDs. This new GQDs synthesis method will allow control of the fundamental properties of materials through intrinsic and extrinsic state tuning, which will in turn allow new devices to be developed with extraordinary properties and functions for numerous applications.

## Conflicts of interest

The authors declare no competing financial interests.

## Supplementary Material

RA-010-D0RA02257H-s001
