# Supplementary material for: Highly luminescent polyethylene glycol-passivated graphene quantum dots for light emitting diodes
Source: RSC Adv. 2020 Jul 22;10(46):27418–23. doi: 10.1039/d0ra02257h (PMC9055582; doi:10.1039/d0ra02257h)
Supplement: RA-010-D0RA02257H-s001 [file RA-010-D0RA02257H-s001.pdf]

Electronic Supplementary Material (ESI) for RSC Advances.
This journal is © The Royal Society of Chemistry 2020

Supporting Information

Highly Luminescent Polyethylene Glycol-passivated Graphene Quantum Dots for Light Emitting Diode

Hyun Jun Kim[a], *Chung Kyeong Lee*[a], *Jin Gwan Seo*[a], *Soon Jik Hong*[a], *Gian Song*[a], *Junghoon Lee*[b,*], *Changui Ahn*[c,*], *DongJu Lee*[c,*], *Sung Ho Song*[a,*]

[a]Division of Advanced Materials Engineering, Kongju National University, Chungnam, Republic of Korea

[b] Divison of Chemical Engineering, Dongseo University, 47, Jurye-ro, Sasang-gu, Busan, Republic of Korea

[c] Engineering Ceramic Center, Korea Institute of Ceramic Engineering and Technology, Icheon, Gyeonggi 17303, Republic of Korea

[d]Department of Advanced Materials Engineering, Chungbuk National University, Chungbuk Republic of Korea

*Corresponding authors. E-mails: shsong805@kongju.ac.kr

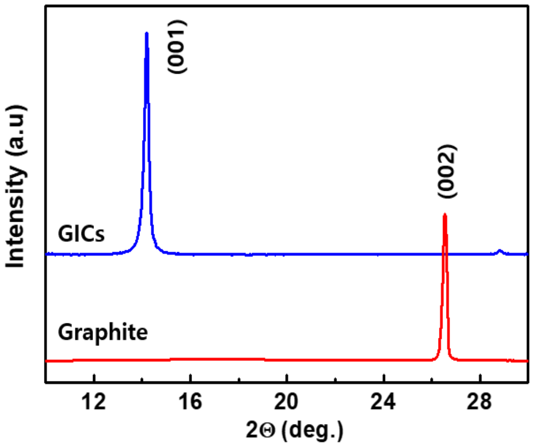

Figure S1. XRD patterns of the GICs (Graphite intercalation compounds) and Graphite

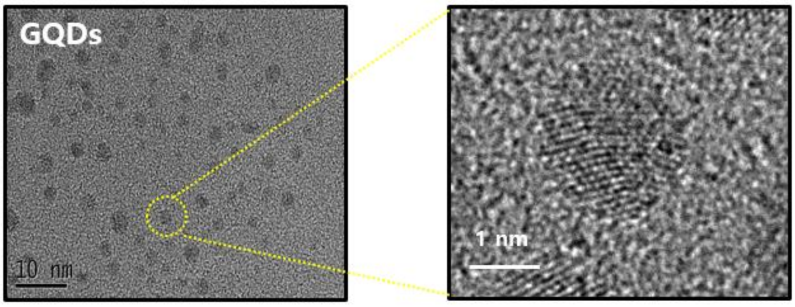

Figure S2. HR-TEM images of GQDs

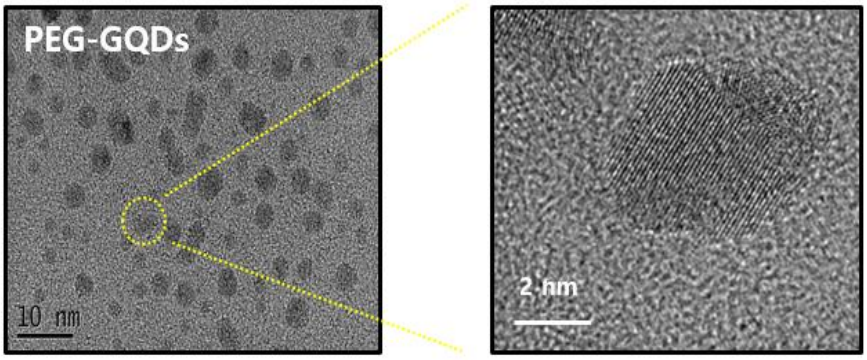

Figure S3. HR-TEM images of PEG-GQDs

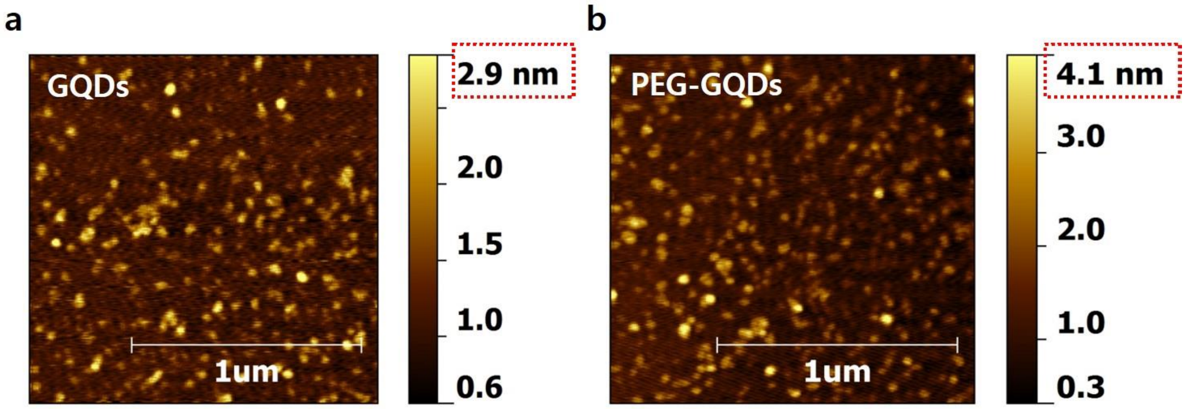

Figure S4. a. AFM image of GQDs. b. AFM image of PEG-GQDs

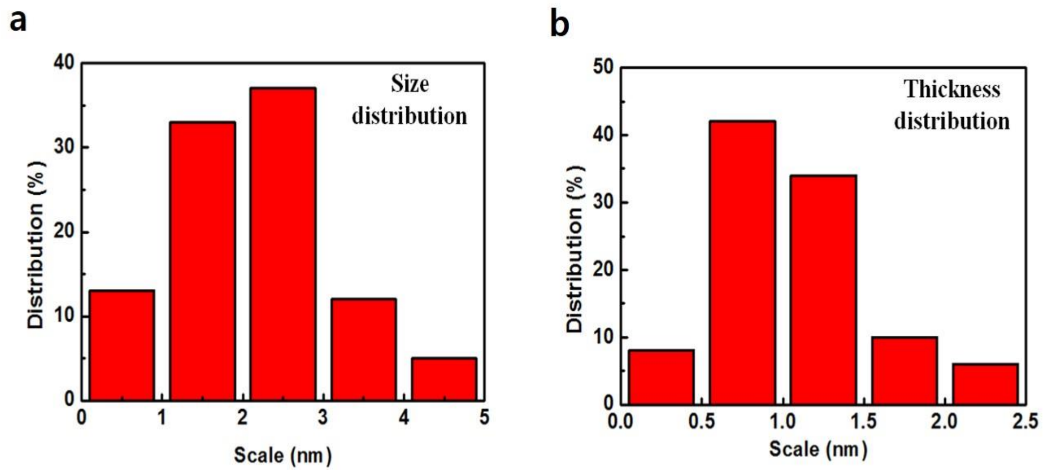

Figure S5. a. Size distribution of the GQDs. b. thickness distribution of the GQDs.

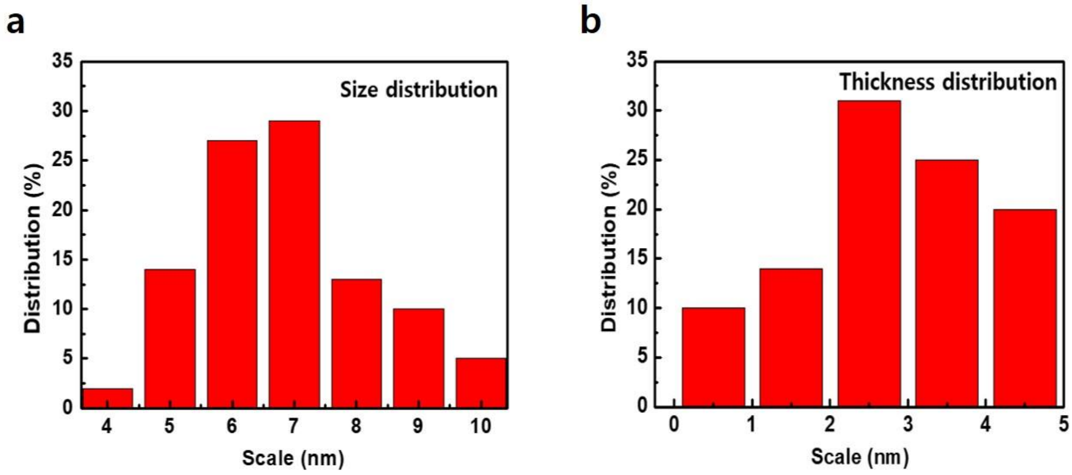

Figure S6. a. Size distribution of the PEG-GQDs. b. thickness distribution of the PEG-GQDs.

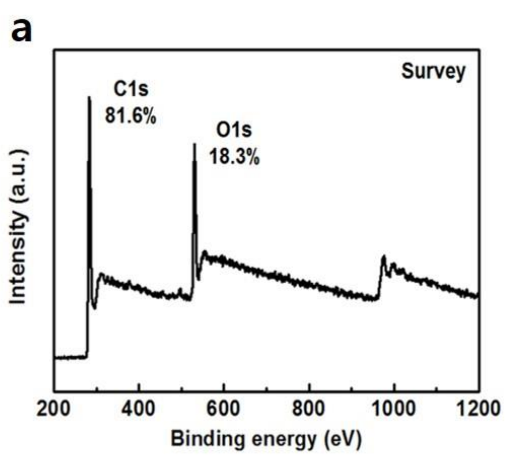

Figure S7. a. High-resolution XPS survey of GQDs. b. High-resolution XPS survey O1s spectra of GQDs.

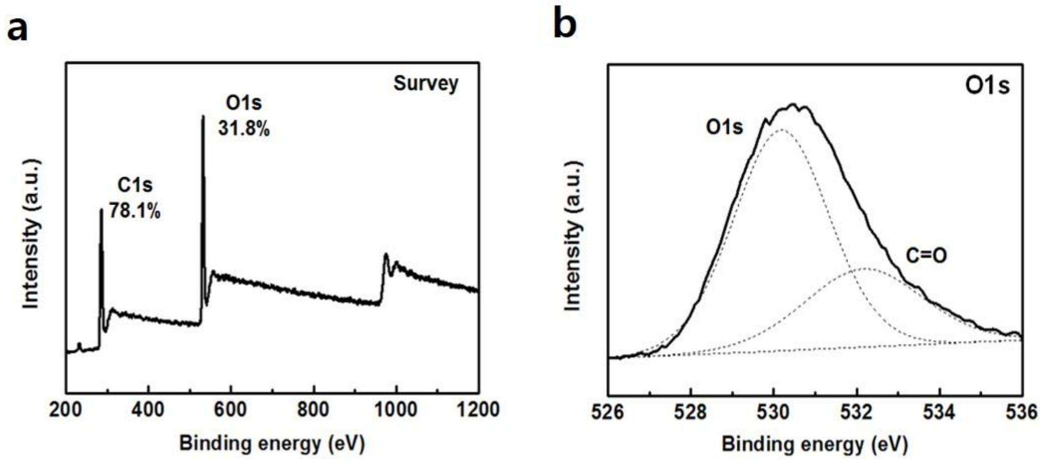

Figure S8. a. High-resolution XPS survey of PEG-GQDs. b. High-resolution XPS survey O1s spectra of PEG-GQDs.

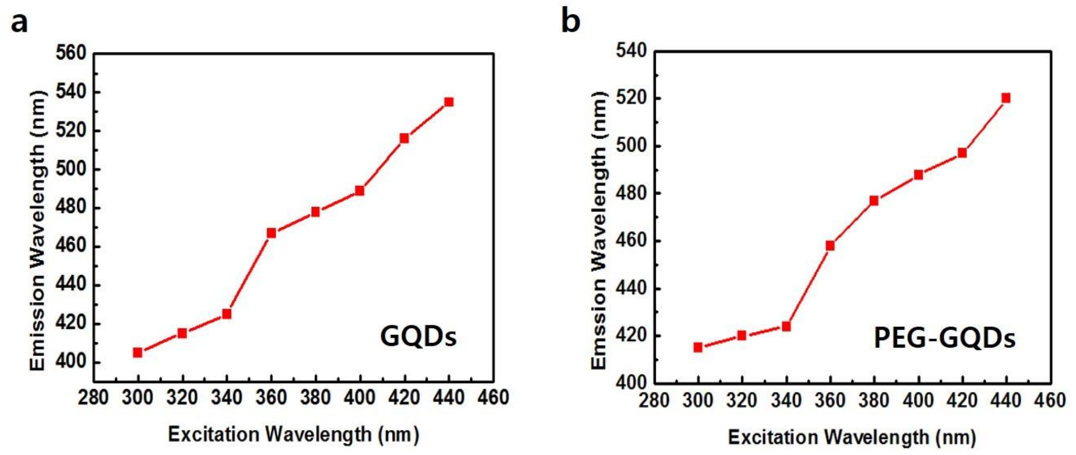

Figure S9. a. PL spectra of GQDs under different excitation wavelength. b. PL spectra of PEG-GQDs under different excitation wavelength.

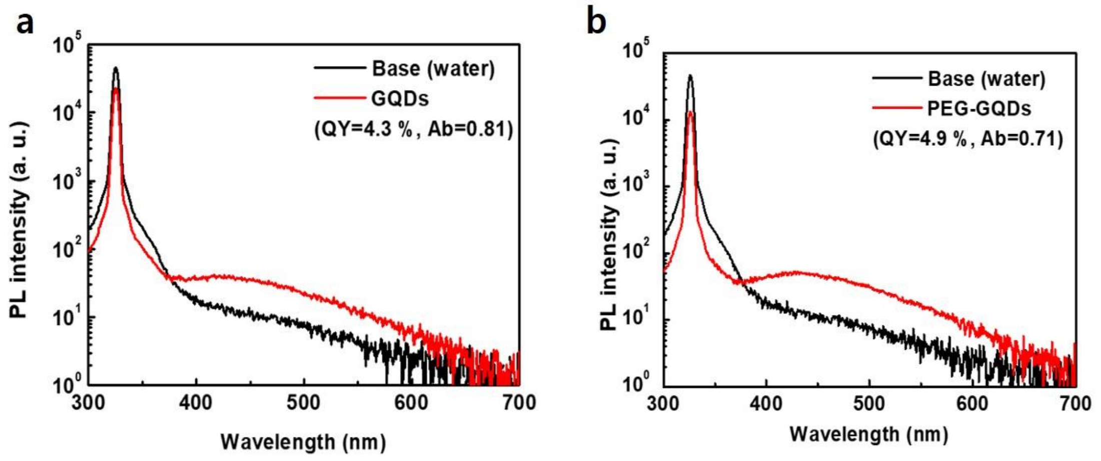

Figure S10. a. Quantum yields of GQDs. b. Quantum yields of PEG-GQDs.

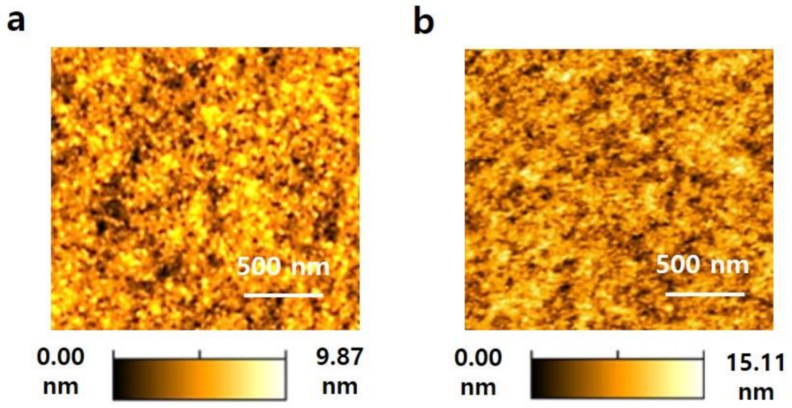

Figure S11. a. AFM images of PVK matrix with GQDs. b. AFM images of PVK matrix with PEG-GQDs.

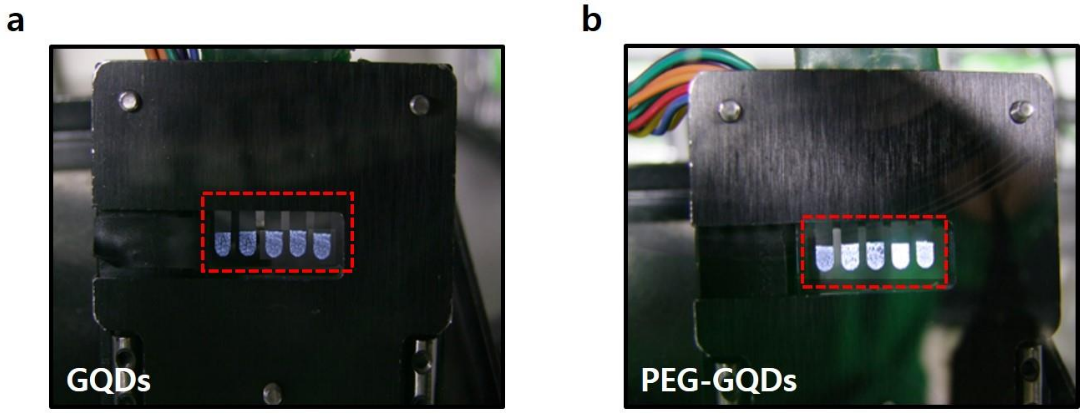

Figure S12. a. Electroluminescent image of GQDs, consisting of five emitting areas. b. Electroluminescent image of PEG-GQDs, consisting of five emitting areas.
